# Mangrove microbiomes: diversity, ecological functions, and applications

**DOI:** 10.3389/fmicb.2026.1908615

**Published:** 2026-09-02

**Authors:** Daniela Muñoz Puebla, Kevin Mindiola-Reyes, Julia Nieto-Wigby, Juan M. Cevallos-Cevallos

**Affiliations:** 1 Escuela Superior Politécnica del Litoral, ESPOL, Centro de Investigaciones Biotecnológicas del Ecuador, Guayaquil, Ecuador; 2 Instituto Nacional de Biodiversidad, Quito, Ecuador; 3 Escuela Superior Politécnica del Litoral, ESPOL, Facultad de Ciencias de la Vida, Guayaquil, Ecuador

**Keywords:** anthropogenic impact, climate change, ecosystem services, mangrove microbiome, microbial diversity, nutrient cycling

## Abstract

Mangrove ecosystems are dynamic coastal environments that offer essential ecological services, socioeconomic benefits, and resilience against climate change. Central to their functionality there are complex microbial communities—primarily bacteria and fungi—that drive key biogeochemical processes such as organic matter decomposition, nitrogen fixation, carbon sequestration, and sulfur cycling. This review aims to synthesize current knowledge on mangrove microbiomes, focusing on microbial taxonomic diversity, ecological roles, and environmental responsiveness. The effect of abiotic factors such as salinity, tidal regimes, vegetation type, and anthropogenic pressures on microbial community structure and function is also assessed. Key findings highlight the presence of both conserved microbial taxa across biogeographic regions and functional adaptations to local conditions, underscoring the global ecological significance of these microbial assemblages. Particular attention is given to microbe-mediated nutrient cycling, symbiotic plant–microbe interactions, and microbial contributions to pollutant degradation, including microplastics and heavy metals. Additionally, recent advances in omics-based approaches have expanded our understanding of microbial functionality and unveiled promising avenues for biotechnological applications, such as enzyme production and bioactive compound discovery. The review also identifies critical knowledge gaps, including the need for long-term monitoring, methodological standardization, and integrated multi-omics frameworks. Overall, recognizing microbial communities as foundational components of mangrove health is essential for effective conservation, ecosystem restoration, and sustainable resource management in the face of accelerating global environmental change.

## 1 Introduction

**Figure 1:**
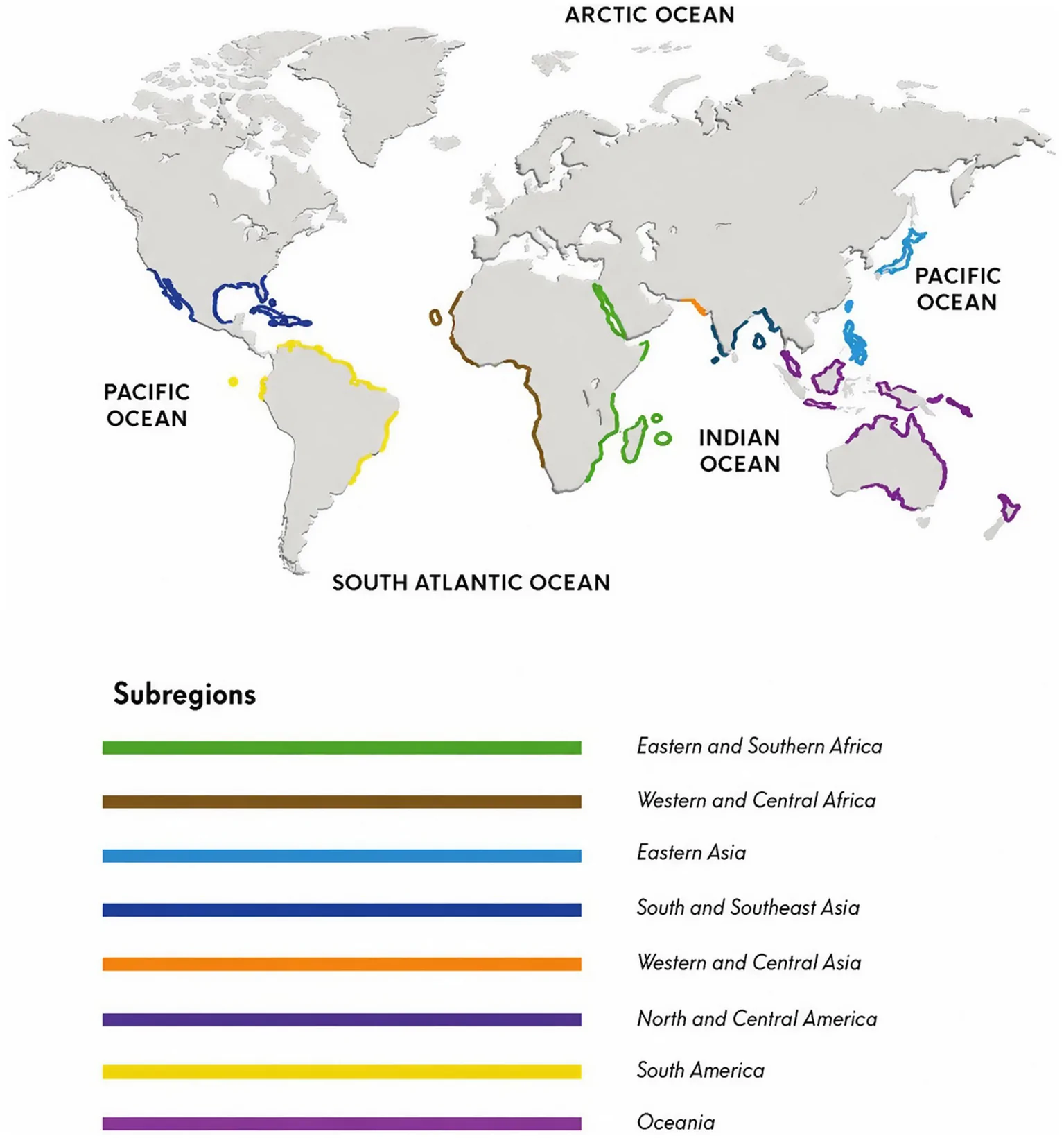
Worldwide distribution of mangroves.

The term *mangrove* is commonly used to refer both to the tree species and to the broader community of organisms that comprise the associated ecosystem (Holguin et al., 2001). These plant formations are usually salt-tolerant and develop in coastal areas of warm or tropical regions, typically characterized by flat, muddy soils and brackish waters, and can even extend inland where salinity decreases. Mangroves exhibit specific adaptations to these environments, such as aerial roots that facilitate gas exchange and structures that excrete excess salt (Charcape-Ravelo and Moutarde, 2005). Mangroves are widespread throughout tropical and subtropical zones, covering approximately 14.8 million hectares globally as of 2020 (Figure 1) and occupying significant portions of coastlines in these regions (FAO, 2023; Fatimah et al., 2023).

Mangrove ecosystems form the foundation of complex trophic networks that support numerous species, many of which hold ecological and economic significance. These environments also provide critical feeding and nursery grounds for a wide range of marine organisms (Holguin et al., 2001) while providing economic resources such as wood, firewood, charcoal, honey, and a variety of medicinal, cosmetic, and other products for the associated communities (Salem and Mercer, 2012). Additionally, mangrove root systems serve as natural buffers, reducing storm impacts and protecting coastlines from erosion (Salem and Mercer, 2012) while enhancing biodiversity and carbon storage, as they sequester atmospheric CO₂ in both above- and belowground structures like leaves and roots (Wainwright et al., 2023; Wirabuana et al., 2025).

Despite their biodiversity richness, mangrove ecosystems are increasingly threatened by climate change and urban pollution (Hau et al., 2024). Between 1996 and 2020, global mangrove cover declined by 3.4% during that period, equivalent to approximately 524,500 hectares, with Southeast Asia being the most affected region, experiencing a reduction of around 245,700 hectares (Leal and Spalding, 2022; Bunting et al., 2022). In general, mangrove cover losses have ranged between 35 and 86% between 1985 and 2010 (Duke et al., 2007; Duke and Larkum, 2008) but recent research shows a recovery since then, with both forest loss and degradation rates slowing over time (Zhang et al., 2026b).

Because many of the mangrove ecosystem functions are mediated or regulated by microorganisms, understanding mangrove-associated bacteria, archaea, fungi, and other microbial groups is essential for explaining nutrient cycling, carbon and sulfur transformations, pollutant degradation, plant resilience, and ecosystem recovery. A deeper comprehension of microbial dynamics can significantly enhance mangrove restoration efforts and support ecosystem resilience under increasing anthropogenic stress (Allard et al., 2020).

This review aims to provide a comprehensive synthesis of current scientific knowledge regarding the microbiome associated with mangrove ecosystems, with particular emphasis on ecological, economic, and social significance of the microorganisms. The study integrates findings from bacterial and fungal diversity, biogeochemical cycling, and the influence of anthropogenic and climatic factors on microbial communities. By focusing on the role of microorganisms in supporting nutrient recycling and habitat resilience, this work seeks to underscore the centrality of the microbiome in the health and sustainability of mangrove forests.

## 2 Review methodology and search strategy

This article was designed as an integrative narrative review rather than a systematic review. Therefore, the literature search was carried out with the objective of synthesizing current knowledge on mangrove-associated microbial communities, their taxonomic diversity, ecological functions, environmental drivers, geographic patterns, responses to anthropogenic stressors, and potential applications. The search focused on peer-reviewed studies reporting bacteria, archaea, fungi, and microbial consortia associated with mangrove sediments, rhizospheres, roots, pneumatophores, leaves, litter, and water-associated compartments.

The search strategy combined targeted keyword searches and backward/forward citation tracking from key review articles and primary studies from peer-reviewed journals in the Scopus or Web of Science databases. Search terms included the word “mangrove” with combinations of: “microbiome,” “microbial community,” “bacteria,” “archaea,” “fungi,” “sediment,” “rhizosphere,” “endophytic fungi,” “actinobacteria,” “16S rRNA,” “ITS sequencing,” “shotgun metagenomics,” “metabarcoding,” “nitrogen fixation,” “denitrification,” “anammox,” “sulfur cycling,” “sulfur cycling,” “sulfate-reducing bacteria,” “sulfur-oxidizing bacteria,” “carbon fixation,” “methane metabolism,” “phosphorus cycling,” “phosphate-solubilizing bacteria,” “microplastic degradation,” “heavy metal bioaccumulation,” “aquaculture,” “urban pollution,” “restoration,” and “Nature-based Solutions.” To further reduce potential recency and selection bias, particular attention was given to studies published between 2024 and 2026 that applied microbiological, amplicon sequencing, shotgun metagenomic, metatranscriptomic, qPCR, or genome-resolved approaches to mangrove-associated samples. These recent studies were summarized separately to identify current methodological trends, dominant microbial taxa, sampling strategies, preservation conditions, DNA extraction protocols, primer sets, sequencing platforms, and analytical workflows used in contemporary mangrove microbiome research (Table 1). This recent-literature synthesis was not intended as a systematic meta-analysis, but rather as a focused update to ensure that the review reflects current methodological practices and recent evidence relevant to microbial diversity and its impact on ecosystem function, conservation monitoring, and restoration assessment in mangrove ecosystems.

**Table 1:** Recent methodological trends in mangrove microbiome studies.

Region of study	Type of study	Brief methodology description	Top dominant taxa	References
Kuwait	Traditional microbiology and NGS	Rhizosphere samples were taken. DNA was isolated using DNeasy PowerSoil Pro Kit (Qiagen) and analyzed by 16S rRNA amplicon (341F/805R) sequencing and isolation of culturable PAH-degrading bacteria.	Traditional microbiology: *Burkholderia, Stenotrophomonas maltophilia, Delftia, Bacillus cereus, Acinetobacter soli,* and *Rhodococcus ruber* NGS dominant groups reported: *Marinomonas*, *Clostridialesbacter*, *Pseudoalteromonas*, *Lactobacillus*, *Sulfurovum*.	Akbar et al. (2026)
Quanzhou Bay, Fujian, China	NGS	Sediments were collected from four mangrove areas, approximately 10 cm depth. 16S rRNA V4 was amplified with 515F/806R; libraries used TruSeq DNA PCR-Free kit and NovaSeq 6000 PE250; QIIME2/DADA2/SILVA were used.	Pseudomonadota, Desulfobacterota, Bacteroidota, Thermoproteota, Acidobacteriota.	Zhang et al. (2026a)
Hainan Island, China;	NGS	Sediment DNA was extracted from 0.5 g sediment using E. Z. N. A. Soil DNA Kit; DNA quality was checked by gel/NanoDrop and stored at −80 °C. 16S V3–V4 was amplified using 338F/806R with FastPfu polymerase.	Proteobacteria, Firmicutes, Actinobacteria, Chloroflexi, Desulfobacterota;	Jiang et al. (2026)
Tamil Nadu, India	NGS	Rhizosphere soils were collected from 15 to 25 cm depth and 1–3 cm from mangrove roots. DNA was extracted using Qiagen Power Soil kit and 16S V3–V4 amplicons were sequenced on Illumina NovaSeq 6000.	Proteobacteria, Actinobacteria, Firmicutes, Fibrobacterota, Chlorobiota, Bacteroidota.	Sujeeth et al. (2026)
Hainan Island, China	NGS	Low-tidal mangrove rhizosphere and bulk sediments were compared with supratidal soils. DNA was extracted from soil samples using the E. Z. N. A.^®^ Soil DNA Kit; 16S rRNA sequencing was used with Tax4Fun2 functional prediction.	Proteobacteria, Chloroflexi, Desulfobacterota, Actinobacteriota, Acidobacteriota, Bacteroidota.	Zhang et al. (2025b)
Shupaisha Island, China	NGS	Seasonal sediment sampling. The top 1 cm was removed; sediment collected with sterile syringe. DNA extraction was with the DNeasy PowerSoil Pro kit (Qiagen), and 16S rRNA sequencing was used with primers 343F/798R.	Proteobacteria, Actinobacteria, Bacteroidetes.	He et al. (2025)
Futian Natural Reserve, Shenzhen, China	NGS + qPCR + metagenomics + metatranscriptomics	Seasonal sediments collected from March 2021 to January 2022. DNA was extracted with DNeasy PowerSoil kit (Qiagen) and applied 16S rRNA (515F/806R) sequencing, metagenomics, and metatranscriptomics.	Proteobacteria, Chloroflexi, Firmicutes, Planctomycetes, Verrucomicrobia, Epsilonbacteraeota, Crenarchaeota, Euryarchaeota.	Zhang et al. (2025a)
Fujian Province, China	NGS + qPCR + shotgun metagenomics + activity assay	Triplicate sediment cores were sliced at 2 cm intervals. DNA extracted from 0.25 g sediment using DNeasy PowerMax Soil Kit (Qiagen); bacterial 16S V3–V4 and archaeal 16S V4–V5 were amplified and sequenced; shotgun metagenomics and metatranscriptomics were also used.	Gammaproteobacteria, Desulfobacteria, Campylobacteria.	Wang et al. (2025)
Sundarbans, India	NGS; shotgun metagenomics	Surface benthic sediment was sampled. DNA was isolated using the conventional phenol–chloroform technique and analyzed by shotgun metagenomics for taxonomic and functional profiling of carbon-cycling genes.	*Paenarthrobacter aurescens*, Roseobacter denitrificans, *Komagataeibacter saccharivorans*, Candidatus *Manganitrophus*, *Halothiobacillus neapolitanus*, *Sphingomonas cynarae*, *Bacillus subtilis*, *Desulforapulum autotrophicum*, *Escherichia coli*, *Pseudomonas* sp., *Roseihalotalea indica*.	Das et al. (2025)
Yucatán Peninsula, Mexico	NGS	Sediments (upper 10 cm) were sampled. DNA was isolated using the DNeasy PowerSoil Kit (Qiagen) 16S rRNA sequencing (515F/806R) was used	Proteobacteria, Desulfobacterota, Chloroflexi, Bacteroidota, Planctomycetota, Gemmatimonadota, Actinobacteriota, Crenarchaeota,	Esguerra-Rodríguez et al. (2024)
Exmouth Gulf, Western Australia;	NGS	Roots fractionated into rhizosphere, rhizoplane, and endosphere were sampled. DNA was extracted using the MagMax Microbiome Ultra Nucleic Acid Extraction Kit (Thermo Fisher Scientific). 16S rRNA sequencing (515F/806R) was used	*Cylindrospermopsis*, Hyphomicrobiales, Rhodobacterales, Ectothiorhodospiraceae	Hsiao et al. (2024)

Studies were included when they provided information on microbial diversity, community structure, functional potential, biogeochemical cycling, plant–microbiome interactions, environmental drivers, geographic variation, anthropogenic disturbance, restoration, or biotechnological applications in mangrove ecosystems. Both culture-dependent and culture-independent studies were considered, including isolation-based studies, 16S rRNA gene sequencing, ITS sequencing, shotgun metagenomics, genome-resolved metagenomics, and functional or biogeochemical studies. Foundational studies were included where historically relevant, while recent omics-based studies were prioritized to reflect the current state of the field.

Studies were excluded when they did not focus on mangroves or clearly mangrove-associated coastal systems, lacked microbial data, addressed only macroecological or botanical aspects without microbial relevance, were duplicated, or consisted of non-scientific web-based material not suitable as primary evidence. Official reports and global assessments were used only for background information on mangrove distribution, conservation status, and ecosystem services.

## 3 Microbial diversity in mangroves

Mangrove ecosystems harbor a rich and complex microbial community that plays a fundamental role in maintaining ecosystem functions. Exploring this microbial diversity is essential for understanding biogeochemical processes, ecosystem resilience, and the potential for biotechnological applications within mangrove environments.

The microbial diversity of mangrove ecosystems exhibits highly variable conditions influenced by extreme gradients of salinity, oxygen availability, pH, and organic matter content in both water and sediment matrices. Furthermore, as interfaces between land and sea, mangroves promote microbial diversity by integrating inputs from both environments. In general, bacteria and fungi constitute 91 % of the total microbial biomass within mangrove ecosystems, while algae and protozoa account for a comparatively minor 7 and 2 %, respectively (Ghizelini et al., 2012).

Microbial studies in mangroves have been carried out using culture-dependent and culture-independent methods. Culture-dependent studies have been used to isolate microorganisms for further evaluation and have mostly been aimed toward isolation of actinobacteria and endophytic fungi. Across studies, actinobacteria—particularly *Streptomyces* and *Micromonospora*—have repeatedly been isolated from soils, rhizospheres, and endophytic compartments (Hong et al., 2009; Sengupta et al., 2015; Jiang et al., 2018; Law et al., 2019; Ye et al., 2023), whereas *Bacillus* and *Pseudomonas* are common among general culturable bacteria (Muwawa et al., 2020; Munir et al., 2025). Similarly, fungal endophyte studies have frequently reported Ascomycota genera such as *Pestalotiopsis*, *Diaporthe*, *Aspergillus*, *Cladosporium*, *Fusarium*, *Alternaria*, *Nigrospora*, and *Xylaria* (Costa et al., 2012; Hamzah et al., 2018; Zhou et al., 2018; Wacira et al., 2020). However, because recovered taxa are strongly shaped by isolation media, tissue type, pretreatment, and incubation conditions, culture-dependent findings should be interpreted as the cultivable and functionally exploitable fraction of mangrove microbiomes, rather than as complete estimates of microbial diversity.

Culture-independent studies of mangrove microbiomes have mostly relied on next-generation sequencing (NGS) with many studies reporting dominant taxa mainly at phylum, class, order, or family level. In contrast to culture-dependent methods, culture-independent analysis has provided a more comprehensive overview of mangrove microbial diversity, primarily through Next-Generation Sequencing (NGS) of marker genes and shotgun metagenomics. Bacterial and archaeal characterization has largely relied on 16S rRNA gene amplicon sequencing, consistently revealing a core microbiome dominated by Proteobacteria (including classes such as Deltaproteobacteria and Gammaproteobacteria), Chloroflexi, Bacteroidetes, and Acidobacteria across diverse geographic regions, from Kenya and India to China, Brazil, and Mexico (Zhang et al., 2017; Dhal et al., 2020; Muwawa et al., 2021; Tavares et al., 2021; Esguerra-Rodríguez et al., 2024). These studies frequently identify key functional groups involved in biogeochemical cycling, such as sulfate-reducing bacteria (e.g., *Desulfovibrio*, *Desulfobacterales*) and various diazotrophic and nitrogen-cycling taxa (Gong et al., 2019; da Costa et al., 2023). Moving beyond taxonomic profiling, shotgun metagenomics has enabled the inference of metabolic functions, confirming the importance of these communities in carbon fixation and methane metabolism (Liao et al., 2020; Mai et al., 2021). Meanwhile, fungal community assessments using Internal Transcribed Spacer (ITS) sequencing have broadly confirmed the dominance of Ascomycota and Basidiomycota, with specific classes like Dothideomycetes, Sordariomycetes, and Eurotiomycetes frequently reported across global mangrove sediments, rhizospheres, and plant tissues (Lee et al., 2019; Zhuang et al., 2020a; Pothayi et al., 2024; Álvarez-Montero et al., 2026).

### 3.1 Bacteria and archaea

Bacteria are one of the most important actors in mangrove ecosystems. The dominant bacterial phyla identified within the rhizosphere include *Acidobacteria, Actinobacteria, Verrucomicrobia*, and the orders *Burkholderiales*, *Caulobacterales*, and *Rhizobiales*, with significantly lower relative abundances observed for *Chloroflexi*, *Firmicutes*, and *Desulfobacterales* (Andreote et al., 2012). Additionally, the isolation of various phosphate-solubilizing bacteria (PSB) including *Bacillus amyloliquefaciens*, *B. licheniformis*, *Enterobacter aerogenes*, *E. taylorae*, *E. asburiae*, and *Kluyvera cryocrescens*, has been reported from the mangrove species *Avicennia germinans* (Holguin et al., 2001).

Culture-dependent techniques have been useful to isolate and characterize bacteria in mangroves. Various taxa including *Bacillus*, *Pseudomonas*, *Micrococcus*, *Microbacterium*, and *Streptomyces*, with species such as *Pseudomonas stutzeri*, *Bacillus cereus*, *B. mycoides*, *Micrococcus luteus*, *Microbacterium paludicola*, and *Streptomyces sanyensis* have been isolated from mangroves in Kenya (Muwawa et al., 2020). Similarly, 29 strains in eight genera and 15 species, including *Aeromonas caviae*, *Bacillus cereus*, *Enterobacter cloacae*, *Klebsiella pneumoniae*, *Myroides profundi*, *Providencia huaxiensis*, *Pseudomonas balearica*, and *Shigella flexneri* were isolated from mangroves in Indonesia (Munir et al., 2025).

Among bacteria, mangrove actinomycetes have frequently been characterized. By using 11 selective isolation media, actinomycetes were isolated from mangrove soils and plant material collected at eight mangrove sites in China, identifying representative bioactive isolates in 13 genera, including *Actinomadura, Microbispora, Nonomuraea, Actinoplanes, Micromonospora, Verrucosispora, Arthrobacter, Isoptericola, Micrococcus, Microbacterium, Nocardia, Rhodococcus,* and *Streptomyces* (Hong et al., 2009). Similarly, seven selective actinomycete media were used to culture actinomycetes from mangrove soil in the Indian Sundarbans, identifying mainly *Streptomyces griseorubens, S. variabilis, S. tendae, S. atrovirens, S. coelicoflavus, and S. lusitanus* (Sengupta et al., 2015). Similarly, 10 isolation media were used to isolate endophytic actinobacteria from leaves, branches, bark, roots, flowers, and fruits of five mangrove plants in China, identifying 101 strains in 28 genera, including *Streptomyces*, *Curtobacterium*, *Mycobacterium*, *Micrococcus*, *Micromonospora*, *Nocardia*, *Nocardiopsis*, and *Verrucosispora* (Jiang et al., 2018). Results are similar to a more recent study with seven selective media used to isolate actinobacteria from rhizosphere soils near mangrove roots in China, obtaining 287 isolates belonging to 10 genera, including *Streptomyces*, *Micromonospora*, *Rhodococcus*, *Sinomonas*, *Nocardia*, *Gordonia*, *Actinomadura*, *Mycobacterium*, *Curtobacterium*, and *Microbacterium* (Ye et al., 2023). Similarly, in Malaysia, 11 culture media, allowed the isolation of 88 putative *Streptomyces* isolates and describing novel species including *Streptomyces monashensis* and *Streptomyces colonosanans* (Law et al., 2019).

Culture-independent methods for microbial characterization of mangroves have relied on NGS. The most-used strategy for NGS bacterial and archaeal characterization has been the use of 16S rRNA gene, identifying dominant Proteobacteria and genera/groups including *Sulfurimonas*, *Arcobacter*, *Woeseia*, *Blastopirellula*, *Spirochaeta*, *Psychrilyobacter*, and *Desulfatitalea* in mangroves from Kenya, with a strong species–environment relationship associated with the levels of calcium, potassium, magnesium, electrical conductivity, pH, nitrogen, sodium, carbon and salinity (Muwawa et al., 2021). In Singapore and Malaysia, the NGS of the 16S rRNA gene allowed identifying a core mangrove microbiome with taxa including *Pleurocapsa*, *Tunicatimonas*, *Halomonas*, *Marinomonas*, *Rubrivirga*, *Altererythrobacter*, *Lewinella*, and *Erythrobacter* (Wainwright et al., 2023). Similarly, mangroves from China showed dominant Deltaproteobacteria and Gammaproteobacteria, diazotrophic groups such as *Desulfobacteraceae, Desulfovibrionaceae, Desulfuromonadaceae, Pseudomonadaceae, Rhodocyclaceae*, and *Comamonadaceae*, and genera including *Desulfovibrio*, *Desulfobacca*, *Desulfobacter*, and *Pseudomonas stutzeri*-related sequences (Zhang et al., 2017), as well as *Chloroflexi, Actinobacteria, Parvarchaeota, Acidobacteria*, and *Cyanobacteria*, with genera including *Desulfococcus*, *Arcobacter*, *Nitrosopumilus*, *Sulfurimonas*, *Roseivivax*, *Erythrobacter*, *Microbulbifer*, *Bacillus*, *Halomonas*, *Vibrio*, *Pseudoalteromonas*, and *Marinobacter* (Gong et al., 2019). A similar study carried out in India identified abundant bacterial families such as *Flavobacteriaceae*, OM1 clade, Oceanospirillaceae, *Spongiibacteraceae*, and several *Rhodobacteraceae* classified as *Nautella* and *Ruegeria* (Dhal et al., 2020); whereas in Brazil, the microbial community was dominated by Deltaproteobacteria, Thermoplasmata, Planctomycetes, Acidobacteria, Bacteroidetes, and Chloroflexi, with indicator genera including *Pontibacillus*, *Halomonas*, *Magnetovibrio*, *Roseimarinus*, *Modicisalibacter*, and functional taxa such as Desulfobacterales and Campylobacterales (Tavares et al., 2021) as well as *Rhizobiales, Rhodospirillales, Rhodobacterales, Desulfobacterales, Syntrophobacterales, Myxococcales, Legionellales, Chromatiales*, and *Methylococcales* (da Costa et al., 2023). Similarly, dominant Proteobacteria, Desulfobacterota, Chloroflexi, Bacteroidota, Planctomycetota, Gemmatimonadota, Actinobacteriota, Crenarchaeota, Acidobacteriota, Spirochaetota, Nanoarchaeota, Cyanobacteria, Myxococcota, Verrucomicrobiota, and Thermoplasmatota were reported in mangroves from Mexico*, with Rhizobiales, Actinomarinales, SBR1031, Desulfobacterales, Desulfatiglandales, Bacteroidales, Anaerolineales, Nitrosococcales, Nitrosopumilales, and Cytophagales* as the main orders (Esguerra-Rodríguez et al., 2024).

Although focusing on the 16S rRNA gene is a widespread strategy for assessing bacteria diversity, the abundance of the original taxa is affected by the PCR amplification, and genus to species identification is not always possible. Shotgun metagenomics attempts to sequence most of the genome of the abundant taxa, so both prokaryotic and eukaryotic microorganisms can be reported while inferring functionality thanks to the various genes detected. For instance, a shotgun metagenomic study carried out in mangroves from Malaysia, identified dominant Gammaproteobacteria, Actinobacteria, Alphaproteobacteria, Deltaproteobacteria, Anaerolineae, Bacilli, and Bacteroidia, with Desulfobacterales and Rhizobiales contributing strongly to carbon fixation, methane metabolism, and related functions (Mai et al., 2021). A similar study carried out in China reported *Nitrospira*, ammonia-oxidizing archaea, sulfate-reducing bacteria and methanogens, as well as reconstructed *Acidobacteria* genomes with numerous biosynthetic gene clusters (Liao et al., 2020).

Bacterial diversity in mangrove ecosystems is influenced by soils under different vegetation types. A study conducted in China reported that aboveground biomass (AGB) exceeded belowground biomass (BGB), and that total forest biomass (TFB) progressively increased with the number of years since vegetation restoration. Analysis through 16S rRNA gene sequencing revealed distinct differences in microbial community composition across soils with varying vegetation cover. In surface soil layers, the dominant bacterial phyla were *Proteobacteria*, *Firmicutes*, and *Chloroflexi*. Within these, *Gammaproteobacteria* emerged as the most abundant microbial class in the surface soil, with relative abundances ranging from 11.41 to 18.85%, followed by *Clostridia* (*Firmicutes* phylum) at 12.84%. The class *Anaerolineae* (*Chloroflexi*) displayed relatively high abundance at unvegetated mudflat (MF) sites (13.63%). The genus *Latescibacter* (phylum *Latescibacterota*) showed significantly higher relative abundance in surface soil samples. In bottom soil layers, *Bacilli* (*Firmicutes*) significantly increased, becoming the most dominant class at site 18LB. Additionally, at unvegetated MF sites, the genera *PLTA13* and *Woeseia* (both belonging to *Gammaproteobacteria*) exhibited significantly greater relative abundance in surface soil compared to subsurface layers (Su et al., 2025). Table 2 presents the stratification of distinct layers in mangrove soils.

**Table 2:** Bacterial communities associated with different layers within mangrove soils.

Compartment/layer	Description	Microbial community	References
Surface soil	Upper soil layer (typically 0–10 cm), rich in organic matter and exposed to oxygen.	*Proteobacteria, Gammaproteobacteria Firmicutes, Chloroflexi, Latescibacter, Planctomycetes, Methanosarcina, Nitrososphaeria, Eurotiomycetes, Ascomycota, Basidiomycota, Glomeromycota*	Su et al. (2025), Ng et al. (2025), and Holguin et al. (2001)
Subsurface layers	Deeper soil layers (below 30 cm), often anoxic and with lower organic content.	*Firmicutes, Proteobacteria, Chloroflexi, Acidobacteria, Spirochaetes, Trichoderma*	Su et al. (2025), Ng et al. (2025)
Aboveground biomass (AGB)	Plant biomass above the soil surface, including leaves, branches, and stems.	*Pseudomonas, Bacillus, Methylobacterium,* diazotrophs *(Rhizobiales), Phyllochora, Pestalotiopsis*	Su et al. (2025) and Yuan et al. (2023)
Belowground biomass (BGB)	Root biomass, both fine and coarse, located below the soil surface.	Rhizobacteria: *Rhizobium*, *Azotobacter, Burkholderia*; Mycorrhizal Fungi	Su et al. (2025), Yuan et al. (2023), and Ng et al. (2025)
Unvegetated mudflat (MF)	Intertidal zone lacking vegetation, typically low in organic carbon and microbial activity.	*Proteobacteria, Anaerolineae (Chloroflexi),* Cyanobacteria PLTA13 and Woeseia (Gammaproteobacteria)	Su et al. (2025)

The impact of shrimp aquaculture on microbial communities in mangrove ecosystems is increasingly recognized as a key indicator of environmental change. Shifts in bacterial composition can reveal how these human activities alter sediment health and nutrient dynamics. A study conducted in Ecuador demonstrated that shrimp aquaculture is linked to increased nitrogen inputs, shifts in microbial community composition, and reduced alpha diversity in mangrove sediments. Differential abundance of taxa such as *Micrococcales*, *Arcobacter*, and *Calothrix* suggests their potential as bioindicators of ecosystem disturbance (Erazo and Bowman, 2021).

### 3.2 Fungi

Fungi are also predominant inhabitants of mangrove ecosystems. The roots of mangrove vegetation release exudates that foster the development of specific microbial associations. Thus, mangrove tissues serve as a reservoir for endophytic fungi (Job et al., 2023) which play a crucial role in sustaining ecosystem productivity and contributing to the overall resilience of the mangrove ecosystem (Gomes et al., 2011).

Fungal diversity in mangrove ecosystems is shaped by interactions between vegetation, plant tissue, and soil structure. The majority of fungal species inhabiting mangrove environments belong to the phyla Ascomycota and Basidiomycota, which dominate many plant-associated fungal communities (Portalanza et al., 2025). However, several members of Ascomycota include parasitic taxa capable of causing plant mortality, contributing to the accumulation of leaf litter, decaying roots, and organic debris. In turn, plant litter supports complex microbial networks involved in decomposition and nutrient cycling (Pan et al., 2018).

Numerous fungal genera have been reported from mangrove species, including *Aspergillus, Aureobasidium, Cladosporium, Curvularia, Cylindrocephalum, Drechslera, Fusarium, Myrothecium, Nigrospora, Penicillium, Pestalotia, Phyllosticta, Trichoderma,* and *Verticillium* (Sarma, 2012). These genera represent a broad taxonomic range within dominant phyla and reflect the complex ecological roles fungi play in mangrove environments.

Different culture media were used to isolate endophytic fungi from mangrove leaf tissues in Malaysia, identifying *Alternaria macrospora*, *Fusarium lateritium*, *Nigrospora oryzae*, *Pestalotiopsis* sp., *Phoma* sp., and *Xylaria* sp. as the main taxa (Hamzah et al., 2018). A similar strategy was applied along the Kenyan coastline, identifying 76 isolates in genera such as *Aspergillus*, *Cladosporium*, *Nigrospora*, *Fusarium*, *Alternaria*, *Lasiodiplodia*, *Chaetomium*, and *Penicillium* (Wacira et al., 2020). Similarly, culture-dependent studies carried out in China characterized endophytic fungi from mangrove roots, stems, leaves, hypocotyls, and flowers, identifying 46 representative isolates across 17 genera, with *Pestalotiopsis* and *Diaporthe* as prevalent taxa (Zhou et al., 2018). Additionally, using culture-dependent methods, 40 taxa of endophytic fungi were reported from Brazilian mangrove leaves, including frequent *Guignardia* sp., *Colletotrichum gloeosporioides*, and *Glomerella cingulate* as well as *Sphaerosporium*, *Chloridium virescens* var. *virescens*, *Microsphaeropsis arundinis, Penicillium pinophilum, Periconia cambrensis, Phoma herbarum, P. diachenii, P. obscurans, Sordaria prolifica* and *Torula elisii* (Costa et al., 2012).

NGS strategies for fungal characterization of mangroves have focused on NGS of the Internal Transcribed Spacer (ITS) DNA region. In Singapore and Peninsular Malaysia, mangrove fungal communities were dominated by Ascomycota/Dothideomycetes, with Agaricomycetes more common in pneumatophores and sediments and Eurotiomycetes in sediments (Lee et al., 2019). Dothideomycetes and Tremellomycetes also dominated the fungal communities in mangroves from China (Yao et al., 2019) as well as *Rozellomycota and Chytridiomycota*, with community assembly affected by geography and sediment depth (Zhang et al., 2021). Similarly, Basidiomycota and Ascomycota were identified as major phyla with dominant classes including *Agaricomycetes, Sordariomycetes, Saccharomycetes, Dothideomycetes,* and *Eurotiomycetes* in India (Haldar and Nazareth, 2019).

Fungal studies have particularly focused on different parts of the mangrove ecosystem (Table 3). Mangrove leaves from Taiwan and China have shown a distinct fungal community dominated by Ascomycota and Basidiomycota with abundant taxa including *Alternaria*, *Cladosporium delicatulum*, *Pyrenochaetopsis leptospora*, *Aureobasidium*, and *Hortaea werneckii* (Chi et al., 2019; Zhu et al., 2023). However, a study on fungal succession during leaf decomposition in sediment–water microcosms from Brazil, showed early dominance of Ascomycota followed by increased Basidiomycota, with *Saccharomycetes, Sordariomycetes,* and *Tremellomycetes* as the predominant classes, as well as plant-pathogen genera including *Pilidiella* and *Pestalotiopsis* (Moitinho et al., 2022). Similarly, ITS amplicon sequencing was applied to analyze fungi from non-rhizosphere, rhizosphere, root episphere, and root endosphere compartments of mangroves in China, showing that Ascomycota and Basidiomycota dominated exterior compartments whereas root endosphere samples had lower fungal richness and more unclassified fungi (Zhuang et al., 2020b). The rhizosphere in mangroves from Kenya was also dominated by Ascomycota and Basidiomycota, with network-associated genera including *Corollospora*, *Moleospora*, *Scedosporium*, and *Aspergillus* (Muwawa et al., 2024). Subsurface mangrove sediments from India, showed Ascomycota as the predominant fungi, with various identified genera, including *Talaromyces*, *Nigrospora*, *Alternaria*, *Candida tropicalis*, *Fusarium solani*, *Rhodotorula mucilaginosa*, *Schizophyllum commune*, and *Bipolaris sorokiniana* (Pothayi et al., 2024) whereas sediment samples from mangroves in Ecuador, were dominated by Ascomycota and Basidiomycota, with common genera including *Ascochyta*, *Antrodia*, *Talaromyces*, *Penicillium*, *Ceramothyrium*, and *Rhizoctonia* (Álvarez-Montero et al., 2026).

**Table 3:** Culture-independent studies of mangrove fungal communities grouped by substrate compartment.

Mangrove substrate compartment	Country/region	Main fungal taxa reported	References
Leaves, fruits, pneumatophores, and adjacent sediments	Singapore and Peninsular Malaysia	Dominated by Ascomycota, especially Dothideomycetes; Agaricomycetes were more common in pneumatophores and sediments, while Eurotiomycetes were more associated with sediments.	Lee et al. (2019)
Leaves, fruits, pneumatophores, and sediments	Peninsular Malaysia	Ascomycota and Basidiomycota dominated; Dothideomycetes were abundant across organs, with Agaricomycetes more common in pneumatophores and sediments.	Lee et al. (2020)
Epiphytic and endophytic fungi from	Southern China	Communities were dominated mainly by Dothideomycetes and Tremellomycetes; 635 fungal OTUs were detected.	Yao et al. (2019)
Leaves	Kinmen County, Taiwan	Ascomycota and Basidiomycota were dominant; abundant taxa included *Alternaria*, *Cladosporium delicatulum*, *Pyrenochaetopsis leptospora*, *Aureobasidium*, and *Hortaea werneckii*.	Gong et al. (2019)
Epiphytic and endophytic fungi from leaves	Qi’ao Island, Guangdong, South China	Ascomycota, Basidiomycota, Cryptomycota, Glomeromycota, and Zygomycota were reported; dominant classes included Dothideomycetes, Tremellomycetes, Eurotiomycetes, and Microbotryomycetes.	Zhu et al. (2023)
Non-rhizosphere soil, rhizosphere, root episphere, and root endosphere	Southern China	Ascomycota and Basidiomycota dominated external compartments; root endosphere samples showed lower fungal richness and a higher proportion of unclassified fungi.	Zhuang et al. (2020b)
Rhizosphere sediments	Gazi Bay and Mida Creek, Kenya	Ascomycota and Basidiomycota dominated; network-associated genera included *Corollospora*, *Moleospora*, *Scedosporium*, and *Aspergillus*.	Muwawa et al. (2024)
Mangrove sediment depth profiles	Eastern to southern China, >9,000 km coastline	High diversity of basal fungal lineages, especially Rozellomycota and Chytridiomycota, with community assembly shaped by geography and sediment depth.	Zhang et al. (2021)
Mangrove sediments	Goa, India	Basidiomycota and Ascomycota were major phyla; dominant classes included Agaricomycetes, Sordariomycetes, Saccharomycetes, Dothideomycetes, and Eurotiomycetes.	Haldar and Nazareth (2019)
Subsurface mangrove sediments	Kannur and Ernakulam, Kerala, India	Ascomycota predominated; reported genera/species included *Talaromyces*, *Nigrospora*, *Alternaria*, *Candida tropicalis*, *Fusarium solani*, *Rhodotorula mucilaginosa*, *Schizophyllum commune*, and *Bipolaris sorokiniana*.	Pothayi et al. (2024)
Mangrove sediments, 10-cm depth	Reserva Ecológica Manglares Churute, Ecuador	Ascomycota and Basidiomycota dominated; 214 genera were detected, including *Ascochyta*, *Antrodia*, *Talaromyces*, *Penicillium*, *Ceramothyrium*, and *Rhizoctonia*.	Álvarez-Montero et al. (2026)
Decomposing leaf material in sediment–water microcosms	Cananéia mangroves, São Paulo, Brazil	Early decomposition was dominated by Ascomycota, followed by increased Basidiomycota; reported classes included Saccharomycetes, Sordariomycetes, and Tremellomycetes, with genera such as *Pilidiella* and *Pestalotiopsis*.	Moitinho et al. (2022)

### 3.3 Factors influencing microbial structure

Several key factors influence the structure of microbial communities in mangrove ecosystems, including tidal levels, vegetation age, soil salinity, pH, total carbon, and sediment depth.

For instance, tidal levels and vegetation age influence the structure of soil microbial communities, exhibiting differential environmental adaptability. Vegetation age in mangrove ecosystems shapes the composition and adaptability of soil microbial communities. As mangrove stands mature, they suffer successive changes that alter root exudation patterns, organic matter inputs, and redox conditions in the sediment. Younger vegetation may support more dynamic microbial assemblages due to fluctuating environmental conditions and lower organic matter stability, whereas older stands tend to harbor more specialized and resilient microbial communities. The study by Su et al. (2025) highlights that phyla *Chloroflexi* and *Proteobacteria* are sensitive to these environmental gradients, whereas *Firmicutes* displayed robust adaptability in the subsoil, particularly at mangrove restoration sites. The class *Bacilli* within *Firmicutes* and the class *Campylobacteria* within the phylum *Campylobacterota* also exhibited strong adaptability in low-tide environmental conditions (Su et al., 2025).

Soil salinity, induced by the intrusion of seawater, also affects the microbial structure in mangroves. Soil pH, total carbon (TC), moisture content, and available particulate nitrogen (APN) have been identified as the primary drivers of bacterial community composition, whereas soil pH and plant characteristics were the principal factors influencing fungal communities in mangroves. The increase in soil salinity and pH, correlating with increasing degradation gradients, significantly accounted for both the increase in the relative abundance of *Actinobacteria*, *Gemmatimonadetes*, *Firmicutes*, and *Deinococcus-Thermus*, and the decrease in *Acidobacteria* and *Nitrospirae* (Wu et al., 2021). Regarding fungal phyla, the abundance of *Zygomycota* and *Glomeromycota* has been shown to decline in response to grassland degradation while other microbial interactions were enhanced, and the study suggested that interspecies associations may facilitate microbial adaptation to harsh soil environments (Wu et al., 2021). In general, fungi have been more sensitive than bacteria to ecosystem disturbance, so the structural composition of soil fungal communities could serve as a bioindicator of grassland health or restoration progress, offering practical applications for post-construction environmental monitoring in ecological rehabilitation projects (Wu et al., 2021). Similarly, mangrove restoration also affects the microbial diversity in the associated ecosystem. In a study conducted in China, the microbial community composition of restored mangrove forests (RMF) and natural mangrove forests (NMF) showed that forest types shared the same dominant taxa. Among prokaryotic communities, *Proteobacteria* was the most abundant phylum, followed by *Bacteroidetes* and *Chloroflexi*. In microeukaryotic communities, *Ochrophyta* and *Cercozoa* were identified as the two predominant phyla. However, notable differences in the relative abundances of these dominant taxa were observed between RMF and NMF. For prokaryotes, *Proteobacteria* and *Chloroflexi* exhibited significantly higher relative abundances in RMF, whereas *Bacteroidetes*, *Planctomycetes*, *Verrucomicrobia*, and *Gemmatimonadetes* were more prevalent in NMF. Similarly, among microeukaryotes, RMF showed increased relative abundances of *Ochrophyta* and *Lobosa*, while *Cercozoa*, *Hilomonadea*, *Fungi*, *Chlorophyta*, *Choanoflagellida*, and *Breviatea* were more abundant in NMF (Zhang et al., 2024). The composition of restored mangrove forests also influences the microbial structure of the mangrove due to plant-microbe interactions and the physical–chemical modifications that different plant species induce in the environment.

**Figure 2:**
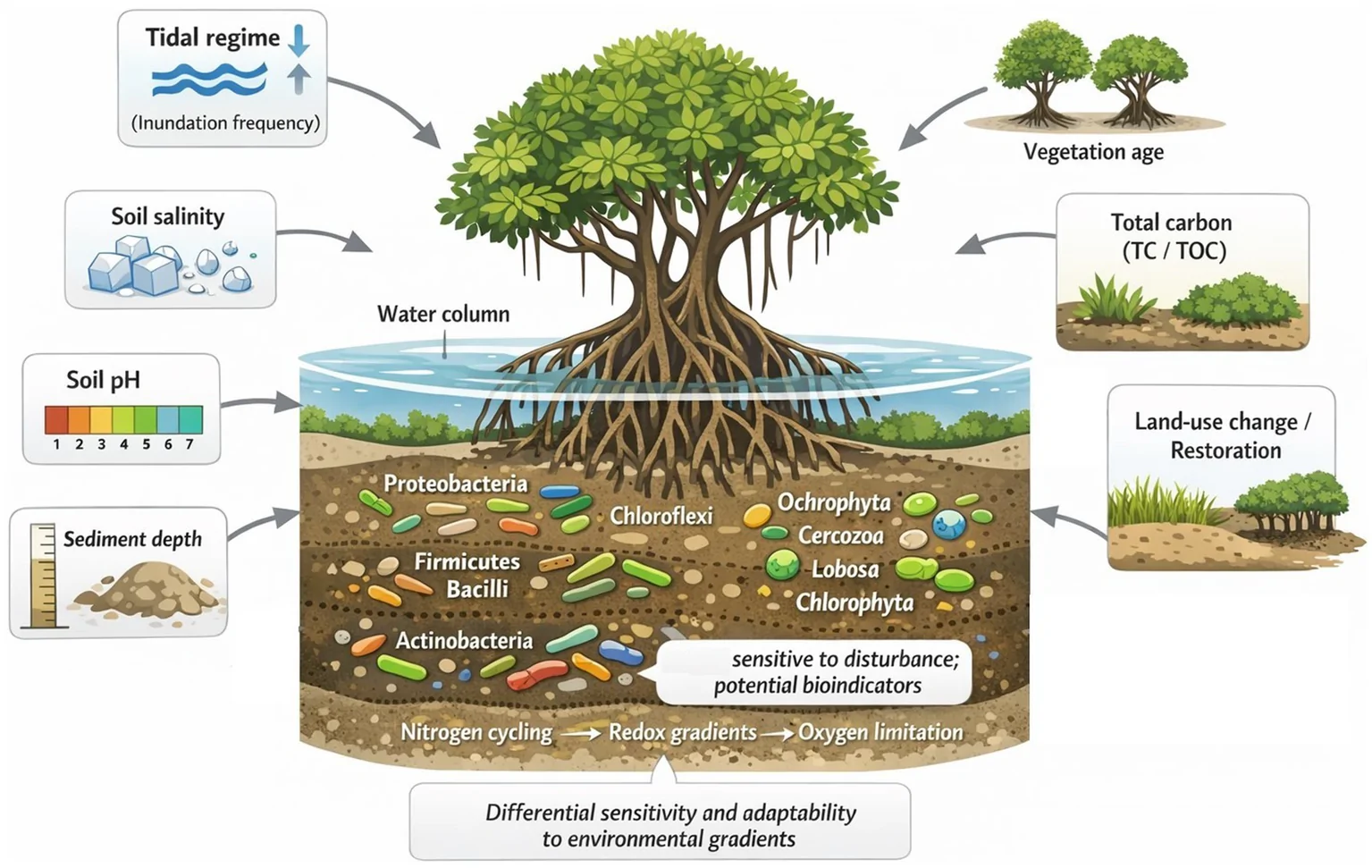
Environmental factors affecting microbial community structure and function in mangrove ecosystems. Details of the prompts provided to the generative AI tool to generate this figure are available in Supplementary material S1.

Sediment depth is a key factor shaping microbial community structure by modifying environmental gradients such as oxygen availability, redox potential, and nutrient concentrations. The inverse relationship between microbial abundance and sediment depth likely reflects declining oxygen and organic matter availability in deeper layers, which constrains microbial activity. In mudflat sediments, nitrogen cycling processes are primarily regulated by pH, total organic carbon (TOC), total sulfur (TS), total nitrogen (TN), and sulfate concentrations, while phosphorus (P) acts as a promoting factor by enhancing microbial-mediated nitrogen transformations, including denitrification and ammonification. In contrast, within mangrove sediments, salinity, TOC, TN, and sulfate emerge as the main drivers stimulating nitrogen cycling, whereas iron (Fe) appears to exert a constraining influence and elevated iron concentrations may inhibit nitrogen transformations by altering redox-sensitive microbial pathways (Yan et al., 2024). Figure 2 illustrates key factors influencing the structure of microbial communities.

## 4 Ecosystem functions of microorganisms in mangroves

### 4.1 Microbial role in biogeochemical processes

Mangrove ecosystems are highly efficient at recycling nutrients, primarily through the decomposition of leaf litter and soil formation processes (Cotrufo et al., 2013). Additionally, mangroves play a significant role in mitigating greenhouse gas emissions and climate change (Mcleod et al., 2011). Microbial communities play a central role in nutrient cycling within mangrove ecosystems. Microorganisms inhabiting root and leaf surfaces enhance micronutrient availability, protect against pathogens, and initiate decomposition during plant senescence (Holguin et al., 2001). In mangrove sediments, microbes mediate key processes such as nitrogen fixation, denitrification, and anaerobic ammonium oxidation (anammox), contributing significantly to nitrogen cycling (Rosentreter et al., 2018).

Microbial decomposition of litter and detritus facilitates the release of nutrients, particularly nitrogen and phosphorus, which support mangrove plant nutrition. Early acetylene-reduction studies provided foundational evidence that biological nitrogen fixation may contribute substantially to mangrove nitrogen demand in some systems, meeting up to 60% of the nitrogen demands of mangrove trees (Zuberer and Silver, 1978). The intertidal nature of mangrove ecosystems—characterized by alternating submergence and anoxic saline soils—may influence root exudation, promoting colonization by nitrogen-fixing bacteria and fostering symbiotic associations with diazotrophs, aka nitrogen fixating microorganisms (Zuberer and Silver, 1978). Core members of the mangrove microbiome involved in nitrogen cycling include *Nitrospiraceae/Nitrospira*, *Rhizobiales incertae sedis/Anderseniella*, and *Rhizobiaceae/Fulvimarina*. Similarly, taxa involved in the sulfur cycle included *Sulfurimonadaceae/Sulfurimonas* and *Sulfurovaceae*/*Sulfurovum* (Wainwright et al., 2023).

Microbial diversity within distinct plant compartments is increasingly recognized for its functional significance. Wainwright et al. (2023) identified eight taxa—*Pleurocapsa*, *Tunicatimonas*, *Halomonas*, *Marinomonas*, *Rubrivirga*, *Altererythrobacter*, *Lewinella*, and *Erythrobacter*—enriched across different mangrove tissues, suggesting key roles in nutrient cycling and host resilience. Members of the core microbiome, such as *Nitrospira* (*Nitrospiraceae*), *Anderseniella* (*Rhizobiales incertae sedis*), and *Fulvimarina* (*Rhizobiaceae*), are closely linked to nitrogen metabolism, while sulfur-cycling taxa like *Sulfurimonas* (*Sulfurimonadaceae*) and *Sulfurovum* (*Sulfurovaceae*) further illustrate the multifunctional microbial support system. Notably, *Pleurocapsa*, a nitrogen-fixing cyanobacterium, is enriched in pneumatophores, indicating a specialized role in nitrogen acquisition under waterlogged, and nutrient-poor conditions (Wainwright et al., 2023).

Bacteria also regulate carbon flow by recycling organic matter and acting as carbon sinks in tropical sediments (Holguin et al., 2001). Metagenomic analysis revealed that Gammaproteobacteria, Desulfobacteria, and Campylobacteria were the most abundant carbon-fixing groups using mainly the Calvin-Benson-Bassham cycle and the Wood-Ljungdahl (WL) pathways (Wang et al., 2025). Additionally, metagenomic functional and taxonomical analysis identified several carbon regulating genes belonging to the mangrove bacterial species *Paenarthrobacter aurescens* TC1, *Roseobacter denitrificans* OCh 114, *Komagataeibacter saccharivorans, Candidatus Manganitrophus* sp.*, Halothiobacillus neapolitanus* c2*, Sphingomonas cynarae, Komagataeibacter saccharivorans, Bacillus subtilis* subsp. *subtilis str.* 168*, Desulforapulum autotrophicum* HRM2, *Escherichia coli* K-12, *Pseudomonas sp*. SG-MS2, and *Roseihalotalea indic*a (Das et al., 2025). Similarly, fungal succession during leaf decomposition showed that fungi such as Saccharomycetes, Sordariomycetes, Tremellomycetes, Eurotiomycetes, and Dothideomycetes; with genera like *Pilidiella* and *Pestalotiopsis* contribute to lignocellulosic litter breakdown (Moitinho et al., 2022).

Mangrove microorganisms have also been reported to significantly participate in sulfur cycling. In general, mangrove sulfur cycling is mainly driven by two interacting guilds: sulfate-reducing bacteria such as *Desulfovibrio, Desulfobacterales, Desulfobacteraceae, Desulfobulbaceae, Syntrophobacteraceae, Desulfosarcina,* and *Desulfatibacillum*, which reduce sulfate to sulfide under anoxic conditions (Balk et al., 2015; Wu et al., 2019; Mo et al., 2021); and sulfur-oxidizing bacteria such as *Thiohalophilus, Chromatium, Thiohalobacter, Sulfurifustis, Allochromatium,* and *Thiobacillus,* which oxidize sulfide, thiosulfate, or elemental sulfur back to sulfate (Li et al., 2021). Other bacteria such as *Micrococcus* spp., *Bacillus* spp., *Pseudomonas* spp., and *Klebsiella* spp. Have been shown to oxidize sulfur compounds and produce sulfate, indicating potential roles in sulfur mineralization and sulfate supply in mangrove soils (Behera et al., 2013).

Mangrove sediment sulfur cycling has been shown to be depth-dependent as surface layers favor sulfur oxidation, DMSP transformation, and sulfur-driven denitrification, whereas deeper anoxic layers favor sulfate reduction, sulfur reduction, and DMS/DMSO transformations (Taketani et al., 2010; Wu et al., 2019; Qian et al., 2023). This vertical partitioning suggests that mangrove sediments operate as tightly coupled sulfur redox systems, where sulfide produced by sulfate-reducing bacteria in deeper layers can be re-oxidized by sulfur-oxidizing microorganisms in surface or root-influenced zones (Li et al., 2021). Therefore, sediment depth, through its effects on redox conditions, pH, acid volatile sulfide, organic matter availability, and sulfate supply, is a major determinant of microbial sulfur-cycle structure and function.

**Figure 3:**
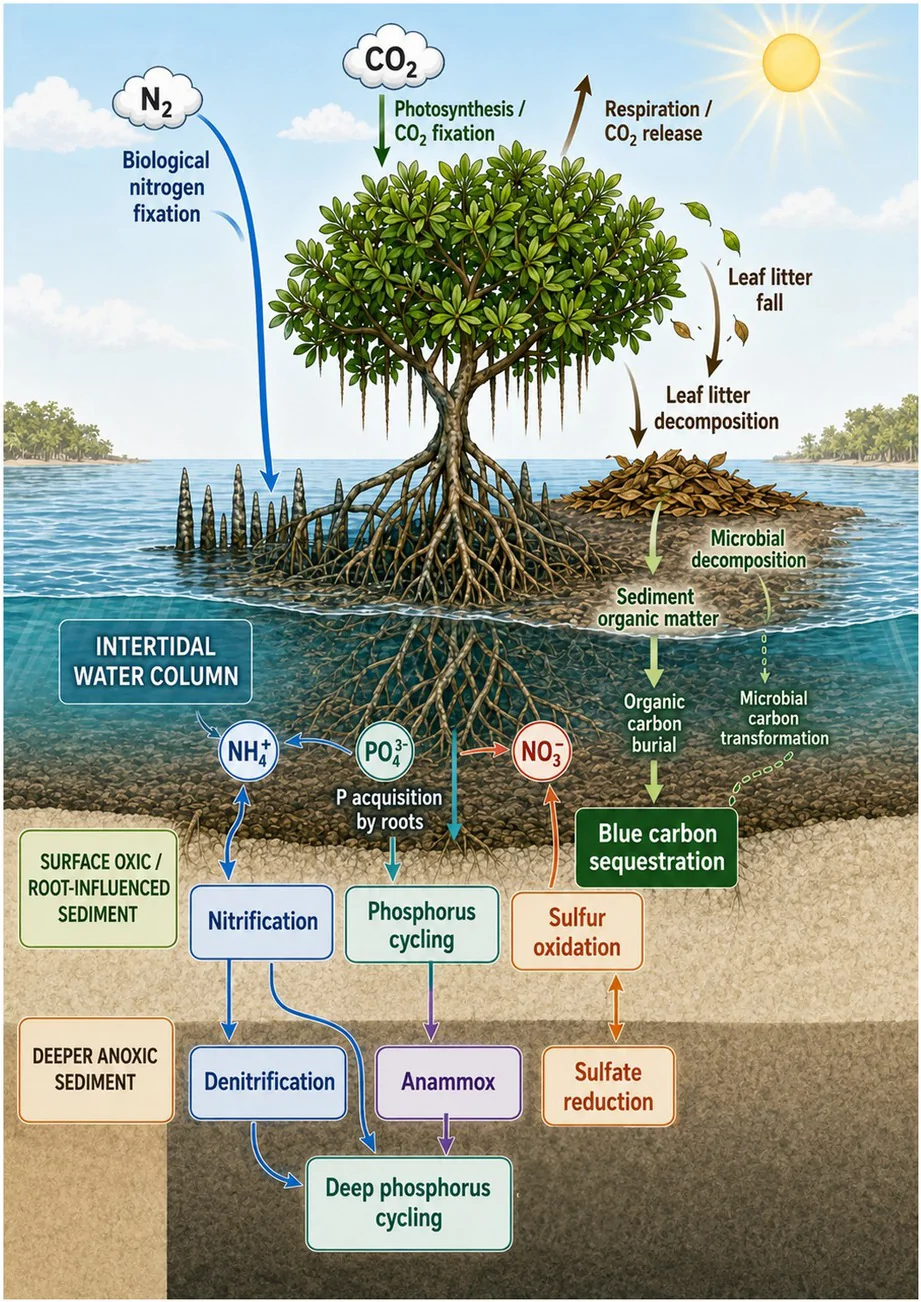
Microbial-mediated carbon, nitrogen, sulfur and phosphorus cycling in mangroves. Details of the prompts provided to the generative AI tool to generate this figure are available in Supplementary material S1.

Phosphorus cycling is also affected by mangrove microbial community. In general, mangrove microorganisms contribute to phosphorus cycling mainly by solubilizing inorganic phosphate, mineralizing organic phosphorus through acid and alkaline phosphatases, and facilitating plant phosphorus acquisition in the rhizosphere through bacterial and mycorrhizal interactions. Culture-based studies repeatedly report *Bacillus, Pseudomonas, Serratia, Alcaligenes, Rhodococcus, Arthrobacter, Enterobacter, Micrococcus, Azotobacter, Lysinibacillus,* and *Glomus* as important phosphorus-mobilizing taxa (Vazquez et al., 2000; Kothamasi et al., 2005; Zhang et al., 2022; Fatimah et al., 2023; Aruna et al., 2024). More recent metagenomic studies show that microbial phosphorus cycling is also depth-dependent with surface sediments mainly acquiring available inorganic phosphorus, whereas deeper sediments rely on hydrolysis of persistent triphosphates, nucleotide recycling, and free-phosphate sourcing (Liu et al., 2025). Additionally, phosphorus and nitrogen cycling in mangroves appear to be functionally connected through glutamate metabolism, suggesting that phosphorus transformations in mangrove sediments are not isolated processes but part of broader nutrient-cycling networks (Liu et al., 2025). Figure 3 illustrates the nutrient cycling microorganisms operating within mangrove ecosystems.

Keystone taxa within mangrove sediments, including sulfate-reducing bacteria such as *Desulfuromonadales*, and sulfur-oxidizing bacteria (SOB), like *Chromatiales*, act as multifunctional organisms that couple nitrogen and sulfur biogeochemical cycles (Huang et al., 2024). Biogeochemical cycles, such as the carbon and nitrogen cycles underscore the essential role of microorganisms as key mediators (Cotrufo et al., 2013). Table 4 summarizes the primary bacterial functions in mangrove ecosystems within the context of biogeochemical cycling.

**Table 4:** Main roles of bacteria in mangrove ecosystems.

Associated biogeochemical cycle	Taxa	Function	References
Nitrogen cycle	Nitrospirae, Rhizobiaceae, *Nitrospiraceae/Nitrospira*, *Rhizobiales incertae sedis/Anderseniella*, and *Rhizobiaceae/Fulvimarina*.	Nitrogen fixation, enhance root development, facilitate nutrient turnover and bioavailability, mediate the degradation of pollutants	Holguin et al. (2001) and Wainwright et al. (2023)
Sulfur cycle	Desulfovibrio, Desulfobacterales, Desulfobacteraceae, Desulfobulbaceae, Syntrophobacteraceae, Desulfosarcina, and Desulfatibacillum, Sulfurimonadaceae Sulfurimonas and Sulfurovaceae Sulfurovum, Desulfuromonadales, Chromatiales	Reduction of sulfate to sulfide, Oxidation of reduced sulfur compounds as an energy source, linking the sulfur and carbon cycles via autotrophic CO₂ fixation.	Wainwright et al. (2023), Huang et al. (2024), Balk et al. (2015), Wu et al. (2019), and Mo et al. (2021)
Carbon cycle	Proteobacteria, particularly Gamma-proteobacteria and Alpha-proteobacteria, Pleurocapsa, Tunicatimonas, Halomonas, Marinomonas, Rubrivirga, Altererythrobacter, Lewinella, and Erythrobacter, *Desulfobacteria*, and *Campylobacteria; Paenarthrobacter aurescens* TC1, Roseobacter denitrificans OCh 114, *Komagataeibacter saccharivorans, Candidatus Manganitrophus* sp.*, Halothiobacillus neapolitanus* c2, *Sphingomonas cynarae, Komagataeibacter saccharivorans*, *Bacillus* subtilis subsp. *subtilis str.* 168*, Desulforapulum autotrophicum* HRM2*, Escherichia coli* K-12*, Pseudomonas* sp. SG-MS2, and *Roseihalotalea indica*; Saccharomycetes, Sordariomycetes, Tremellomycetes, Eurotiomycetes, and Dothideomycetes; with genera like *Pilidiella* and *Pestalotiopsis*	Carbon fixation and degradation. Crucial role in cycling of dissolved organic matter in aquatic systems	Wang et al. (2025), Das et al. (2025), and Moitinho et al. (2022)
Phosphorus cycle	*Pseudomonas, Serratia, Alcaligenes, Rhodococcus, Arthrobacter, Micrococcus, Azotobacter, Lysinibacillus, and Glomus, Bacillus amyloliquefaciens, B. licheniformis, Enterobacter aerogenes, E. taylorae, E. asburiae*, and *Kluyvera cryocrescens*	Regulates phosphorus availability, degrades organic compounds containing phosphorus, releasing inorganic phosphate (P) into the environment.	Vazquez et al. (2000), Kothamasi et al. (2005), Zhang et al. (2022), Fatimah et al. (2023), Aruna et al. (2024), and Holguin et al. (2001)

Beyond taxonomic composition, microbial functional activity in mangrove ecosystems is strongly structured by habitat type and sediment depth. In unvegetated mudflats, aerobic ammonia oxidation predominates in surface sediments, whereas nitrification, dark sulfide and sulfur oxidation, and aromatic compound degradation are more prevalent in deeper layers. In contrast, vegetated low-tide mangrove sites exhibit enhanced surface-layer activities such as methanol oxidation, chitinolysis, phototrophy, photoheterotrophy, and nitrite ammonification, while anammox activity is concentrated in subsurface sediments (Su et al., 2025). This vertical stratification highlights the strong coupling between environmental gradients and microbial functional potential in intertidal systems.

While microbial processes sustain ecosystem productivity, they are highly sensitive to environmental disturbance. Microbial diversity and functional composition can therefore serve as sensitive bioindicators of ecosystem stress (Alongi, 1994). In mangrove systems affected by aquaculture effluents, shifts toward sulfur-oxidizing bacteria, sulfate-reducing bacteria, denitrifiers, and potential pathogens have been reported, reflecting reduced nutrient-use efficiency and increased pathogenic potential. Heavily disturbed sites also exhibit transitions from nitrogen-fixing to denitrifying taxa, indicating imbalances in nitrogen cycling dynamics (Erazo and Bowman, 2021).

Restoration strategies, particularly mangrove reforestation, have been proposed to enhance microbial functionality and ecosystem resilience. Reforested mangroves facilitate the re-establishment of microbial, plant, and animal communities, promoting recolonization by keystone taxa such as diazotrophs and other microorganisms essential for biogeochemical cycling (Wirabuana et al., 2025).

As a result, reforested mangroves function as highly efficient blue carbon sinks, capturing atmospheric CO₂ in dense aboveground biomass and in anoxic sediments where organic matter decomposition is suppressed. Their high primary productivity supports strong trophic dynamics and carbon retention, ultimately enhancing ecosystem resilience and contributing to regional and global biogeochemical balance.

### 4.2 Plant-microbiome interaction

Mangroves inhabit some of the most physiologically demanding habitats on Earth, enduring dynamic salinity regimes, anoxic sediments, and limited nutrient availability. The exceptional plant resilience and elevated productivity under these extreme conditions are not just the result of intrinsic physiological traits but are fundamentally sustained by complex and essential interactions with diverse microbial consortia. The mangrove microbiome is profoundly linked to plant-derived processes and exudates; inhabits plant compartments, sediments, water column; and produces biofilms on mangrove roots (Holguin et al., 2001).

Plant–microbiome interactions in mangroves are particularly important for plant growth and nutrient acquisition. Among the microbial groups involved, plant growth-promoting rhizobacteria (PGPR) play a central role in enhancing nutrient cycling and stress tolerance. Taxa belonging to *Corynebacteriales*, *Burkholderiales*, and *Rhizobiales*, have been reported as key PGPR in mangrove ecosystems (Huang et al., 2024). Further inoculation with the keystone strain *Novosphingobium* sp. significantly enhanced nitrogen fixation rates (NFR) and nitrogen retention in seedling-associated sediments. Moreover, successful colonization by this strain actively shaped the surrounding microbial landscape, facilitating the assembly of a more complex and functionally enriched microbial community (Huang et al., 2024).

Mangrove microbiome has also been associated with the efficient cycling of organic carbon and nitrogen. In general, surface soils have shown active carbon cycling (methanol oxidation, chitinolysis), while deeper layers were dominated by nitrogen cycling (Su et al., 2025). Microbial degradation of chitin—a significant component of plant and fungal biomass—is crucial for remineralizing organic matter and liberating essential carbon and nitrogen resources that sustain the broader microbial community and facilitate the continuous turnover of detritus in wetlands (Dedysh, 2023). Furthermore, the robust metabolism of specific carbon compounds, such as methanol, significantly enhances soil organic carbon content in mangrove ecosystems. The enrichment of methanol oxidation genes has suggested greater microbial use of this carbon source, supporting specialized communities (Su et al., 2025).

Nutrient availability further modulates microbial community composition and function across sediment layers. In surface soils, ammonium (NH₄^+^-N) and microbial biomass carbon (MBC) emerge as significant nutrient drivers, likely influenced by plant root exudates and active nitrogen metabolism. In contrast, nitrate (NO₃^-^-N) and soil bulk density are primary regulators of microbial activity in subsoils, where nitrifying and denitrifying microorganisms dominate (Su et al., 2025). However, salinity consistently exerts a strong inhibitory effect across both surface and subsurface strata, disproportionately affecting salinity-sensitive taxa and constraining microbial functional diversity (Su et al., 2025).

Beyond bacteria, fungal communities also exhibit specialized ecological strategies within mangrove ecosystems. Recent studies from Isla Santay (Ecuador) revealed a stratified and diverse fungal assemblage associated with *Cyperus rotundus*, highlighting the ecological complexity of fungal–plant interactions in sensitive coastal environments. A prominent finding is the consistently high relative abundance of *Cladosporium dominicanum* in rhizospheric samples. This observation aligns with previous reports identifying *Cladosporium* species as ubiquitous colonizers of a broad spectrum of plant substrates, ranging from healthy tissues to decomposing organic matter. Their simultaneous presence in both above- and belowground plant compartments indicate a flexible ecological strategy that may involve endophytic behavior, saprotrophic activity, or a facultative combination of both, depending on environmental conditions (Portalanza et al., 2025). The coordinated activity of bacterial and fungal communities—both finely regulated by nutrient availability and abiotic stressors such as salinity—highlights the deeply integrated nature of plant–microbe interactions.

In general, comprehensive assessments integrating biotic factors (e.g., plant taxonomy, anatomical traits, benthic fauna) with key abiotic drivers (e.g., salinity, temperature, tidal dynamics, and pollution) are still required to fully elucidate the structure and functional dynamics of mangrove microbiomes (Allard et al., 2020).

### 4.3 Additional microbial functionalities

Beyond their central roles in productivity and nutrient cycling, mangrove-associated microorganisms provide critical ecosystem services and hold strong biotechnological potential. Many microorganisms mediate pollutant transformation or produce bioactive compounds, underscoring their ecological and added value. For example, mangroves trap large amounts of microplastics (MPs), a growing global pollutant. Some bacterial and fungal strains from these ecosystems can degrade MPs, highlighting their potential for sustainable bioremediation (Talukdar et al., 2023). Similarly, fungal genera like *Aspergillus, Absidia, Cunninghamella, Mucor,* and *Rhizopus* can bioaccumulate heavy metals, offering low-cost alternatives for detoxifying contaminated sediments (Cardoso et al., 2010).

Mangrove microorganisms are also valuable for drug and enzyme discovery. Endophytic fungi such as Nigrospora parvum and Biscogniauxia spartinae have shown antibacterial activity with low cytotoxicity, suggesting promising applications in medicine and biotechnology (Cadamuro et al., 2021).

More broadly, bacteria inhabiting mangrove ecosystems contribute to multiple ecosystem functions, including enhancement of root development, facilitation of nutrient turnover and bioavailability, and degradation of organic and inorganic pollutants (Holguin et al., 2001; Jiang et al., 2026). Collectively, these multifunctional microbial activities position mangrove microbiomes as key contributors to ecosystem resilience and as valuable biological resources for biotechnological innovation.

### 4.4 Challenges in protocols for mangrove microbiome analysis

Cheung et al. (2018), Cunill-Flores et al. (2023), Rubio-Piña and Zapata-Pérez (2011), Das et al. (2013), Rubio-Piña and Zapata-Pérez (2011), Das et al. (2013), and Sahu et al. (2012) Recent mangrove microbiome studies are dominated by culture-independent approaches, particularly 16S rRNA amplicon sequencing, ITS metabarcoding, shotgun metagenomics, metagenome-assembled genome reconstruction, qPCR, and, in fewer cases, metatranscriptomic or activity-based assays (Table 1). These studies have been conducted across diverse geographic contexts, and have analyzed sediments, rhizospheres, roots, root compartments, leaves, mudflats, among others (Esguerra-Rodríguez et al., 2024; Hsiao et al., 2024; Zhang et al., 2025a; Das et al., 2025; He et al., 2025; Zhang et al., 2025b; Wang et al., 2025; Sujeeth et al., 2026) with sampling depth ranging from surface sediments to cores of 90–100 cm. Sample preservation methods included transport on ice, storage at −20 °C or −80 °C, Longmire’s solution, DNA/RNA Shield, or immediate processing, and DNA extraction protocols mostly used PowerSoil/PowerSoil Pro kits, E. Z. N. A. Soil DNA Kit, FastDNA SPIN Kit for Soil, or phenol–chloroform extraction. Similarly, PCR and sequencing strategies varied across studies, with common bacterial primer sets targeting 16S rRNA V3–V4 or V4 regions, including 338F/806R, 341F/805R or 806R, and 515F/806R, whereas fungal studies commonly used ITS2 primers such as fITS7/ITS4 (Ng et al., 2025; He et al., 2025; Jiang et al., 2026). Although a clear methodological trend is observed in recent studies, there are protocol variations that highlight the importance of clearly reporting sampling design, compartment type, sediment depth, preservation conditions, DNA extraction method, inhibitor-removal steps (if applied), primer sets, sequencing platform, controls, bioinformatic pipeline, and taxonomic database to improve comparability and reduce selection or methodological bias across mangrove microbiome studies.

High concentrations of tannins and other secondary metabolites in mangrove tissues has traditionally complicated the extraction of high-quality nucleic acids, limiting molecular analyses of plant–microbe interactions (Cunill-Flores et al., 2023). Troubleshooting steps have included rapid sample preservation in liquid nitrogen or nucleic acid stabilization buffer, mechanical disruption using bead-beating, use of CTAB- or commercial soil/plant DNA extraction methods modified for polyphenol-rich samples, addition of polyvinylpyrrolidone or polyvinylpolypyrrolidone to bind polyphenols, inclusion of β-mercaptoethanol or other reducing agents to limit oxidation (Rubio-Piña and Zapata-Pérez, 2011; Das et al., 2013), and additional purification steps to remove humic acids, polysaccharides, salts, and PCR inhibitors (Sahu et al., 2012). However, recent studies have increasingly relied on commercial soil or environmental DNA extraction kits, such as PowerSoil/PowerSoil Pro, E. Z. N. A. Soil DNA, without the need of additional tannin or humic acids removal steps (Table 1). Commercial kits usually include DNA-binding, washing, elution, and inhibitor-removal steps; and are therefore expected to reduce co-extracted humic substances, salts, and other PCR inhibitors commonly present in mangrove sediments and rhizosphere samples. In this case, only PCR products are usually further purified using magnetic beads such as the Agencourt AMPure XP magnetic beads to remove residual primers, primer dimers, enzymes, and small PCR artifacts prior to NGS (Esguerra-Rodríguez et al., 2024; Das et al., 2025; He et al., 2025; Akbar et al., 2026; Sujeeth et al., 2026).

## 5 Geographic patterns of microbiomes in mangroves

The composition and functional potential of mangrove-associated microbial communities are not uniform across the globe; instead, they exhibit significant regional variations influenced by a complex interplay of geographic, environmental, and host-specific factors. Understanding the geographic impact of microbial communities in mangroves is crucial for developing targeted conservation and restoration strategies.

Mangrove ecosystems in Southeast Asia—which account for 34% of global mangrove cover—showcase remarkable microbial diversity and functional adaptations (Romañach et al., 2018). Studies in Southern Taiwan revealed a high abundance of fungi, including *Aspergillus* spp. and *Penicillium* spp., with *A. niger* being notably prevalent, alongside dominant bacterial phyla such as *Proteobacteria*, *Actinobacteria*, *Planctomycetes*, and *Chloroflexi*. These patterns were often correlated with salinity levels, indicating its significant influence on community structure (Purahong et al., 2019). Further west, Malaysian mangroves were dominated by Proteobacteria, Firmicutes, Bacteroidetes, Chloroflexi, Actinobacteria, Planctomycetes, and Cyanobacteria. However, functionality analysis in managed productive forests characterized by significantly higher total organic carbon, showed an overabundance of genes related to carbohydrate metabolism, especially those for plant cell wall polysaccharide degradation when compared to pristine mangrove areas (Priya et al., 2018). Similarly, Metagenomic analysis of sediment isolates from Kerala, India showed strong taxonomic similarities at higher divisions, extending to the phylum level, with Euryarchaeota dominating Archaea (Imchen et al., 2017). Carbon sequestration potential varied in Indian seagrass beds within mangrove proximity, highlighting the importance of management for enhanced carbon storage (Mishra et al., 2023).

Across Southeast Asia, taxa like *Pleurocapsa*, *Halomonas*, and *Marinomonas* were enriched in mangrove tree compartments (Wainwright et al., 2023) whereas in Hong Kong, the prevalence of *Candida/Lodderomyces* clade yeasts, and a potentially novel *Yamadazyma* species, was observed (Hau et al., 2024). Consistent patterns emerged from China, where three Proteobacteria subclasses (Delta-, Alpha-, and Gammaproteobacteria) were broadly distributed, with Gammaproteobacteria often exceeding 25% of all phyla (Li et al., 2019). High-abundance bacterial populations, including *Neisseria*, *Pseudomonas*, and *Desulfotomaculum*, were also found (Meng et al., 2022). Furthermore, Proteobacteria consistently dominated mangrove leaf microbiomes across different species, with Bacteroidetes also being prominent (Elyamine et al., 2023). Similarly, in Saudi Arabia (gray mangrove) Proteobacteria, Bacteroidetes, and Firmicutes were the most dominant bacterial phyla (Alzubaidy et al., 2016).

Moving to other global regions, both commonalities and unique adaptations of mangrove microbiomes become apparent. In East Africa, bacterial communities dominated across three regions (83.8%), with *Proteobacteria*, *Bacteroidetes*, *Firmicutes*, and *Planctomycetes* consistently prevalent (Rampadarath et al., 2018). However, the unexpected diversity in fish-associated microbiomes from the African coast suggests significant undocumented microbial diversity linked to dynamic mangrove habitats (Bragança et al., 2021).

In the Americas, studies reveal similar microbial roles alongside region-specific traits. In Florida, USA, high rates of nitrogen fixation were observed, particularly correlated with organic matter in sediments, and occurring without significant lag phases under both aerobic and anaerobic conditions, suggesting wide adaptability of diazotrophs to the intertidal stress (Zuberer and Silver, 1978). Mangrove species in Mexico exhibited varied antioxidant activities, with *Rhizophora mangle* showing higher activity than *Avicennia germinans* (Cunill-Flores et al., 2023).

In South America, Brazilian mangroves from São Paulo revealed varying abundances of *Bacteroidetes*, *Chloroflexi*, and *Planctomycete*s across different zones, underscoring local environmental influences (Andreote et al., 2012). While overall carbon stocks in Brazilian mangroves were high, soil carbon reserves were relatively low compared to above-ground biomass (Kauffman et al., 2018). In Ecuador, *Aspergillus niger* and *A. aculeatus*, fungi also found in Asian mangroves, were identified in restored sites, suggesting common fungal colonizers across geographically distant regions (Sacheri-Viteri et al., 2021). Finally, in Colombia, endophytic bacteria from *Rhizophora mangle* demonstrated high salt tolerance (up to 15–20% NaCl), proteolytic and amylolytic activities, and atmospheric nitrogen fixation, reinforcing their potential for promoting plant growth and reforestation efforts in saline environments (Rolong, 2022). A schematic overview illustrating microbial exchange between world regions is provided in Table 5.

**Table 5:** Environmental drivers and microbial responses by region.

Region	Environmental drivers	Dominant microbial taxa	Observed microbial response/correlation	References
Asia (southern Taiwan, Malaysia, China, Hong Kong, Saudi Arabia, Kerala)	Salinity (NaCl concentration), Total Organic Carbon (TOC) - higher in managed forests	*Proteobacteria*, *Actinobacteria, Planctomycetes, Chloroflexi. Aspergillus* spp. (*A. niger*, *A. flocculosus*), *Penicillium* spp. (*P. steckii*, *P. coffeae*, *P. citrinum*), *Proteobacteria Bacteriodetes Firmicutes, Candida, Ascomycota*	Significantly correlated with microbial community composition. Managed forests (higher TOC) show overabundance of genes for plant cell wall polysaccharide degradation; distinct microbial communities.	Purahong et al. (2019), Priya et al. (2018), Li et al. (2019), Meng et al. (2022), Elyamine et al. (2023), Hau et al. (2024), Alzubaidy et al. (2016), and Imchen et al. (2017)
East Africa (Mozambique, Madagascar, Tanzania, Somalia)	Salinity (NaCl concentration), tidal variation, high variability of oxygen, nitrate and nitrite ammonification	*Proteobacteria, Gammaproteobacteria, Actinobacteria, Bacteroidetes, Firmicutes, Planctomycetes, Archaea, Burkholderiaceae, Planctomycetaceae, Rhodobacteraceae,* and *Desulfobacteraceae*	High representation of microbial taxa involved in sulfate and nitrogen reduction, as well as methanogenesis	Rampadarath et al. (2018)
Oceania (Australia, New Zealand, Fiji, Papua New Guinea)	Salinity (NaCl concentration), seasons, temperature, tidal variation, turbidity, chlorophylla, pH, DO (Dissolved oxygen)	*Proteobacteria, Planctomycetes, Chlamydiales, Desulfobacteraceae, Enterococcaceae, Bacteroidetes, Nitrospirales, Acidobacteria, Firmicutes, Cyanobacteria*	Microbial communities are shaped by higher sulfur content and reducing conditions, favoring sulfate-reducing taxa. Oxidized and oligotrophic environment supports aerobic or nitrifying bacteria, whose abundance is known to decline under anoxic conditions.	Parks et al. (2013) and Kaestli et al. (2017)
North America (Florida USA)	Salinity (NaCl concentrations),mangrove root systems, presence of organic material (leaf fragments), intertidal stress (alternating immersion/exposure, anoxic sediments), Nitrogen addition	*Chromatium, Epsilonbacteraeota, Arcobacter, Sulfurimonas, Marinobacter, Ignavibacterium, Desulfobacterales, Nitrococcus*	Nitrogen fixation correlated with organic matter; predisposition to colonization by nitrogen-fixing bacteria due to stress.	Zuberer and Silver (1978) and Craig et al. (2021)
South America (Brazil, Colombia, Ecuador)	Salinity (NaCl concentrations), pH, organic Matter, temperature, oxygen, nitrate availability, vegetation recovery.	*Proteobacteria, Bacteroidetes, Firmicutes, Actinobacteria, Cyanobacteria, Acidobacteria, Clostridia, Desulfobacteraceae, Chloroflexi Planctomycetes, Aurantimonas endophytica, Mangrovicella endophytica, Mangrovibrevibacter kandeliae, Aspergillus niger, A. aculeatus, Cladosporium dominicanum*	The low-oxygen and organic-rich conditions of mangrove sediments create a favorable niche for anaerobic microbes, including sulfate reducers and methanogens	Rolong (2022), Andreote et al. (2012), Sacheri-Viteri et al. (2021), and Portalanza et al. (2025)

Across the diverse global distribution of mangrove ecosystems, several consistent patterns emerge in their associated microbial communities, often dominated by bacterial phyla such as Proteobacteria, Bacteroidetes, and Firmicutes. This suggests a core set of microbial groups universally adapted to mangrove conditions. However, significant regional and local variations exist in the abundance of specific genera and fungi, as well as in the prevalence of certain functional genes (e.g., carbohydrate metabolism in managed Malaysian sites, methanol oxidation in Chinese mangroves, and nitrogen fixation in Florida and Colombia). These differences are intrinsically linked to localized environmental factors, including salinity gradients, organic matter content, management practices, and specific plant host species, which exert strong selective pressures shaping the microbial inhabitants.

The consistent finding of stress-tolerant and bioremediation-capable microbes (e.g., salt-tolerant bacteria, heavy metal bioaccumulating fungi, microplastic degraders) across continents further emphasizes the adaptive capacity of the mangrove microbiome to varied anthropogenic and natural stressors. Continued geographically-resolved research using standardized methodologies is crucial to fully elucidate the global drivers of mangrove microbiome structure and function, which will in turn inform more effective, regionally tailored conservation and restoration strategies for these invaluable blue carbon ecosystems. Future mangrove microbiome studies would benefit from standardized sampling and reporting protocols to improve comparisons across regions, vegetation types, sediment depths, and disturbance gradients. Ideally, studies should report geographic coordinates, mangrove species, vegetation age or restoration status, tidal level, sediment depth, salinity, pH, redox potential, organic carbon, total nitrogen, total phosphorus, sulfate, moisture content, and contamination status where applicable. Sampling should distinguish plant compartments, including leaves, roots, pneumatophores, rhizosphere, bulk sediment, and water column, and should include depth-stratified sediment sampling because microbial functions vary strongly across oxic and anoxic layers. Technical metadata should include DNA extraction method, primer set, sequencing platform, bioinformatic pipeline, taxonomic database, quality-control parameters, and use of negative and positive controls. Such standardization would improve cross-study comparability and support geographically resolved models of mangrove microbial structure and function. The selection of the omics tool will depend on the objective of the study. For broad taxonomic profiling, 16S rRNA gene sequencing for bacteria and archaea and ITS sequencing for fungi remain cost-effective and widely comparable approaches. However, amplicon sequencing provides limited functional resolution. Shotgun metagenomics is more informative for identifying functional genes involved in nitrogen, sulfur, carbon, and phosphorus cycling, whereas metatranscriptomics can identify actively expressed pathways under specific environmental conditions. Metabolomics and stable isotope approaches can further validate whether predicted pathways correspond to actual biogeochemical activity. Therefore, future studies should ideally combine amplicon sequencing for community structure, shotgun metagenomics for functional potential, and targeted activity measurements or geochemical data for process validation.

## 6 Impact of human interventions on mangroves’ microbial communities

Given the close ties between mangroves and human wellbeing, the ongoing global decline of mangroves highlights the urgent need to better understand their microbiomes (Allard et al., 2020). Although the rate of mangrove loss slowed by 44%—from 18.2 to 10.2 thousand hectares per year between 2000 and 2010 and 2010–2020—the decline continues (FAO, 2023), underscoring the importance of advancing ecological and microbial research. In general, human impact on mangroves is associated with the increasing demand for aquaculture (Erazo and Bowman, 2021) and increase in urbanization (Henderson et al., 2020), affecting the microbial diversity in mangrove ecosystems. Undisturbed mangrove sites harbor communities with larger genomes and greater metabolic versatility, including taxa like *Singulisphaera acidiphila* (*Planctomycetes*), suggesting generalist strategies that support ecosystem resilience. In contrast, disturbed sites show reduced microbial and metabolic diversity, smaller genomes, and fewer 16S rRNA gene copies—trends associated with ecosystem degradation (Erazo and Bowman, 2021). Additionally, the disturbed mangrove sites showed sharp declines in prokaryotic biomass (threefold lower) and heterotrophic production (fivefold lower), along with losses in sensitive meiofaunal groups such as *Cladocera*, *Kinorhyncha*, *Priapulida*, and *Tanaidacea*. Such shifts indicate habitat degradation and weakened ecosystem function (Carugati et al., 2018).

Microbial indicators have proven useful for assessing anthropogenic impacts on mangrove ecosystems, particularly from aquaculture. For example, *Pseudomonas balearica*—previously associated with contaminated wetlands—has been proposed as a bioindicator of disturbed mangrove environments (Salvá-Serra et al., 2017). Similarly, photosynthetic sulfur bacteria (PSB) from the *Chromatiaceae* family have shown promise as indicators of agricultural effluent contamination (Mohd-Nor et al., 2018). Overall, aquaculture-driven disturbance alters microbial communities, favoring sulfur-oxidizers, sulfate reducers, denitrifiers, and potential pathogens—microbes linked to impaired nutrient cycling and decreased ecosystem resilience (Erazo and Bowman, 2021). These findings emphasize the urgent need for sustainable land-use strategies to preserve mangrove health and maintain the critical ecosystem services they provide.

## 7 Influence of microorganisms on mangrove responses to environmental change

Mangrove ecosystems are increasingly exposed to multiple environmental stressors—including pollution, sea-level rise, coastal development, and rising sediment salinity—that threaten ecosystem structure and microbial functioning. Among these pressures, salinity fluctuations are particularly impactful, as they disrupt mangrove physiology and strongly reshape associated microbial communities. Nevertheless, certain halotolerant microorganisms can mitigate salinity stress through mechanisms such as compatible solute production and ion transport regulation, contributing to ecosystem resilience. Investigating these microbial responses, particularly through controlled laboratory and mesocosm experiments, enables precise assessment of stressor-specific effects on mangrove microbiomes (Allard et al., 2020). Understanding and anticipating how global change affects the mangrove microbiome represents a significant challenge, but also a valuable opportunity for the protection, management, and mitigation of impacts on these at-risk ecosystems (Allard et al., 2020). However, challenges associated with this growth—such as pollution and declining water quality—necessitate innovative strategies that align environmental and economic incentives to mitigate ecosystem service loss and restore previously degraded areas (Goto et al., 2023).

In addition to responding to environmental stressors, mangrove-associated microorganisms can actively influence the capacity of mangrove plants and sediments to tolerate and recover from environmental change (Allard et al., 2020; Huang et al., 2024; Soldan et al., 2019). Beneficial rhizosphere and endophytic microorganisms may enhance nutrient acquisition, particularly nitrogen and phosphorus availability, promote root development, and contribute to plant establishment under saline and anoxic conditions (Holguin et al., 2001; Wainwright et al., 2023; Huang et al., 2024). Halotolerant bacteria can support mangrove adaptation to salinity stress through osmoprotectant production, ion homeostasis, and modulation of plant stress responses (Allard et al., 2020). Likewise, microbial communities involved in carbon, nitrogen, sulfur, and phosphorus cycling help maintain sediment fertility, regulate redox conditions, and support organic matter stabilization in anoxic sediments (Holguin et al., 2001; Rosentreter et al., 2018; Wainwright et al., 2023; Su et al., 2025; Huang et al., 2024). These functions are especially relevant in degraded or restored mangroves, where the recovery of microbial functional diversity may contribute to ecosystem resilience, blue carbon storage, and long-term restoration success (Allard et al., 2020; Wirabuana et al., 2025; Mai et al., 2021).

Mangroves also play a key role in greenhouse gas (GHG) emissions. Anaerobic sediments support methanogenesis by archaea, while nitrifying and denitrifying bacteria produce nitrous oxide (N₂O). Microbial metabolism along redox gradients drives both GHG production and consumption, suggesting mangroves may act as net sources of methane (CH₄) and N₂O. Understanding these microbial processes is essential for accurate carbon and nitrogen budgeting and for developing climate mitigation strategies (Rosentreter et al., 2018).

Mishra et al. (2023), Allard et al. (2020), and Goto et al. (2023) beyond their ecological role, mangroves and their microbiomes also offer promising solutions for environmental remediation. Studies of mangrove sediments in Malaysia identified multiple bacterial strains with the capacity to biodegrade microplastics, including species within the class *Bacilli* (e.g., *Bacillus cereus*, *B. gottheilii*, *B. pseudomycoides*) and γ-Proteobacteria (e.g., *Acinetobacter schindleri*, *Stenotrophomonas maltophilia*). These bacteria showed ability to degrade polyethylene (PE), polypropylene (PP), polyethylene terephthalate (PET), and polystyrene (PS) as the sole carbon source in aqueous media (Auta et al., 2017). Although plastic degradation rates by mangrove microbiome were moderate (between 1.6 and 7.4% plastic degradation in 40 days), these findings underscore the potential of mangrove-derived bacteria for bioremediation strategies (Auta et al., 2018).

The concept of microbial restoration holds considerable promise for enhancing the recovery and long-term sustainability of degraded mangrove ecosystems. Targeted interventions, such as the inoculation of beneficial microorganisms or the manipulation of environmental conditions to favor the establishment of resilient microbial communities, can accelerate the restoration of key ecosystem functions. Exploring the potential of microbial inoculation and biostimulation strategies offers innovative approaches to address the challenges facing mangrove conservation in the face of ongoing environmental change.

Microbiome-informed conservation and restoration have the potential to emerge as a promising strategy to complement traditional restoration activities. In this scenario, microbial conservation and restoration of mangroves should be adapted to the dominant environmental pressures and ecological functions of each mangrove region, rather than applied as a uniform strategy. In aquaculture-impacted mangroves, such as those reported in Ecuador, restoration monitoring should prioritize microbial indicators of eutrophication, anoxia, altered nitrogen cycling, and pathogen enrichment, including shifts from nitrogen-fixing taxa toward denitrifiers, sulfur-cycling bacteria, and disturbance-associated groups. In reforested or hydrologically restored mangroves, microbial monitoring should focus on the recovery of diazotrophs, sulfate-reducing and sulfur-oxidizing bacteria, methanotrophs, carbon-cycling microorganisms, and other keystone taxa involved in nutrient turnover, sediment stabilization, and blue carbon accumulation. Similarly, in regions where salinity, tidal regime, sediment depth, or vegetation age strongly structure microbial communities, conservation strategies should include standardized sampling across plant compartments, sediment depths, and physicochemical gradients to distinguish natural microbial variability from degradation-driven shifts. Therefore, microbiome data can complement vegetation cover, sediment chemistry, and hydrological indicators by providing early-warning markers of ecosystem stress and functional recovery.

## 8 Mangroves as nature-based solutions

Mangroves are a source of Nature-based Solutions (NbS) to provide climate resilience, pollution mitigation, and ecosystem services. Notably, NbS linked to mangrove ecosystems have shown the highest success rates in risk mitigation (80%), when compared to forests (77%) and other coastal ecosystems (73%) (Vicarelli et al., 2024). Beyond physical protection, mangrove ecosystems harbor diverse microbial communities with biotechnological applications. These microbiomes contribute to nutrient cycling, carbon sequestration, and plant health (Allard et al., 2020; Soldan et al., 2019). Therefore, mangroves offer dual benefits as ecosystem services like nutrient retention and water purification can be accompanied by other ecological benefits including microbial applications (Lovelock et al., 2024; Goto et al., 2023).

Microbial bioprospecting from mangroves has the potential to provide a sustainable income source. Beyond their ecological and economic functions, mangrove ecosystems represent a promising source of microorganisms with valuable biotechnological capabilities, including the production of enzymes, proteins, antibiotics, and salt-tolerant genes (Mamangkey et al., 2021). Microorganisms, in general, are crucial for enzyme production due to their efficiency and genetic versatility, including amylases, proteases, cellulases, and chitinases, which have applications across multiple industries. The exploration of mangrove microbial strains is still in its early stages, but recent studies have identified *Vibrio* species with the potential to solubilize phosphates (Mamangkey et al., 2021), as well as *Bacillus* strains capable of producing α-amylases (Kanimozhi et al., 2014) with diverse applications, including environmental pollutant removal, detergent and paper synthesis, alcohol production, and use in the textile and baking industries (Schallmey et al., 2004). Similarly, *Bacillus amyloliquefaciens* isolated from mangroves of India exhibited significant antimicrobial activities against a range of pathogenic bacteria, including *Aeromonas caviae*, *Vibrio parahaemolyticus*, and methicillin-resistant *Staphylococcus aureus* (Rajan et al., 2021).

Other mangrove microorganisms contribute to nutrient cycling, carbon sequestration, and plant health. For instance, beneficial bacteria from *Avicennia marina* propagules enhance root development and stress tolerance in plants (Allard et al., 2020; Soldan et al., 2019). Additionally, several mangrove bacteria exhibit salinity tolerance that could be leveraged to improve agricultural resilience under climate stressors. In Malaysia, differential abundance patterns of microbial taxa—such as higher levels of Alphaproteobacteria, Deltaproteobacteria, and Bacilli in *Rhizophora apiculata* and *Bruguiera parviflora*, and elevated Gammaproteobacteria in *Sonneratia alba*—reflect complex ecological interactions in mangrove systems. Notably, *R. apiculata* sediments, characterized by greater microbial diversity and higher total organic carbon (TOC), also exhibited an increased presence of genes linked to carbon metabolism, underscoring the role of mangrove-associated microbiomes in supporting carbon sequestration and ecosystem productivity (Mai et al., 2021). In general, these findings underscore the significant potential of mangrove bacteria for the biotechnology industry.

## 9 Future perspectives and conclusion

Mangroves represent ecosystems of high ecological, strategic, and scientific value. Their relevance transcends the environmental sphere, also encompassing social and economic dimensions, particularly in regions such as Ecuador, where coastal communities directly depend on the ecosystem services these systems provide. However, these communities face increasing pressures derived from industrialization, pollution, and other forms of anthropogenic disturbance, which necessitates integrative approaches that promote both local empowerment and the generation of validated scientific knowledge.

The mangrove-associated microbiome remains an active area of exploration. Microbial species—bacteria, fungi, and other taxa—play fundamental roles in key ecological processes, such as nitrogen, carbon, and sulfur cycling. The literature demonstrates that these processes are intimately linked to the metabolic activities of specific microorganisms cohabiting with mangrove plant species, generating synergies that enhance the ecological resilience of the ecosystem. Furthermore, comparative geographical analyses reveal how distinct regions present edaphic, physicochemical, and biological variations that sculpt the structure and functionality of mangrove microbiomes. These differences can be attributed to local environmental factors, as well as to the influence of anthropogenic impacts and management strategies implemented in each zone. Despite advancements in molecular biology, particularly in the taxonomic identification of microbial species via omics techniques, there remains a critical need for research contextualized within natural mangrove conditions, in addition to studies that delve deeper into the functional and metabolic characterization of these microorganisms, which will allow for a more precise understanding of their ecological role and their potential to strengthen the adaptive capacity of mangroves in the face of global change.

In this context, mangrove microbiomes also emerge as Nature-Based Solutions (NbS), adding to the intrinsic mangrove’s potential for climate change mitigation, coastal protection, and the provision of essential ecosystem services. Mangroves harbor microbial communities with notable biotechnological potential. These associations can be applied in sectors such as agriculture, through the promotion of root development, enhancement of plant establishment, and increased tolerance to salinity conditions, a critical factor under scenarios of climate stress. The reviewed studies illustrate how certain mangrove-associated bacteria participate in metabolic pathways that underpin key ecological processes within sediments, thereby reinforcing their role in the global productivity of the ecosystem.

Overall, mangroves should not be understood merely as coastal ecosystems, but rather as fundamental pillars of sustainability in a world increasingly pressured by environmental challenges. Their microbial richness encapsulates a vast, underexplored potential, whose scientific evidence and understanding require greater knowledge accessibility and integrated strategies for conservation, management, and sustainable utilization. Recognizing and acting upon this potential is essential to ensure their preservation and to harness their multifaceted long-term benefits.
