# Supplementary material for: Mangrove microbiomes: diversity, ecological functions, and applications
Source: Front Microbiol. 2026 Sep 2;17:1908615. doi: 10.3389/fmicb.2026.1908615 (PMC13579365; doi:10.3389/fmicb.2026.1908615)
Supplement: Supplementary file 1 [file Supplementary_file_1.docx]

**Prompt for figure 2:**

Create a professional scientific figure for the attached review article draft. The figure should show a central mangrove ecosystem in cross-section, including mangrove trees, aerial roots, water column, rhizosphere, and sediment layers. Surround the mangrove with the major environmental drivers that influence microbial communities, illustrated with scientific icons and connected to the mangrove by arrows.

Environmental drivers to include are those mentioned in the attached draft, including: Tidal regime (inundation frequency), Vegetation age, Soil salinity, Soil pH, Total carbon (TC/TOC), Land-use change and mangrove restoration, and Sediment depth

Within the sediment, depict representative microbial groups and their spatial distribution according to the draft:

Bacteria such as Proteobacteria, Chloroflexi, Firmicutes (including Bacilli), Actinobacteria; Microeukaryotes including Ochrophyta, Cercozoa, Lobosa, Chlorophyta; and Fungal communities as bioindicators of disturbance

Show environmental gradients and ecological processes occurring in sediments, including: Nitrogen cycling, Redox gradients, Oxygen limitation with increasing depth

Use a clean scientific illustration style suitable for a peer-reviewed review article. Employ a white background, professional typography, muted natural colors, vector-style graphics, and clear directional arrows linking environmental drivers to microbial community structure and function. The overall message should emphasize that interconnected environmental gradients regulate microbial diversity, adaptability, and ecosystem processes in mangrove ecosystems.

**Prompt for figure 3:**

Create a scientific-style digital illustration of nutrient cycling in mangrove ecosystems based on the information from the attached review article draft. The figure should center on a mangrove tree with aerial roots and pneumatophores in an intertidal coastal setting. Include the atmosphere, water column, oxic and anoxic sediments, leaf litter, roots, and microbial communities mentioned in the draft. Show arrows for CO₂ uptake through photosynthesis, biological nitrogen fixation from N₂, leaf litter decomposition, carbon recycling, nutrient release as NH₄⁺ and PO₄³⁻, denitrification and anammox, methane-related processes, sulfur cycling, and blue carbon sequestration as described in the draft. Include representative microbial taxa and use a didactic, clean, publication-ready style suitable for a scientific review article
